# Adaptive decision-making depends on pupil-linked arousal in rats performing tactile discrimination tasks

**DOI:** 10.1152/jn.00309.2022

**Published:** 2023-11-15

**Authors:** Shreya Narasimhan, Brian J. Schriver, Qi Wang

**Affiliations:** Department of Biomedical Engineering, Columbia University, New York City, New York, United States

**Keywords:** adaptive decision-making, behavioral adaptation, drift diffusion model, Go/No-go tactile discrimination task, pupil-linked arousal

## Abstract

Perceptual decision-making is a dynamic cognitive process and is shaped by many factors, including behavioral state, reward contingency, and sensory environment. To understand the extent to which adaptive behavior in decision-making is dependent on pupil-linked arousal, we trained head-fixed rats to perform perceptual decision-making tasks and systematically manipulated the probability of Go and No-go stimuli while simultaneously measuring their pupil size in the tasks. Our data demonstrated that the animals adaptively modified their behavior in response to the changes in the sensory environment. The response probability to both Go and No-go stimuli decreased as the probability of the Go stimulus being presented decreased. Analyses within the signal detection theory framework showed that while the animals’ perceptual sensitivity was invariant, their decision criterion increased as the probability of the Go stimulus decreased. Simulation results indicated that the adaptive increase in the decision criterion will increase possible water rewards during the task. Moreover, the adaptive decision-making is dependent on pupil-linked arousal as the increase in the decision criterion was the largest during low pupil-linked arousal periods. Taken together, our results demonstrated that the rats were able to adjust their decision-making to maximize rewards in the tasks, and that adaptive behavior in perceptual decision-making is dependent on pupil-linked arousal.

**NEW & NOTEWORTHY** Perceptual decision-making is a dynamic cognitive process and is shaped by many factors. However, the extent to which changes in sensory environment result in adaptive decision-making remains poorly understood. Our data provided new experimental evidence demonstrating that the rats were able to adaptively modify their decision criterion to maximize water reward in response to changes in the statistics of the sensory environment. Furthermore, the adaptive decision-making is dependent on pupil-linked arousal.

## Graphical abstract

**Figure:**
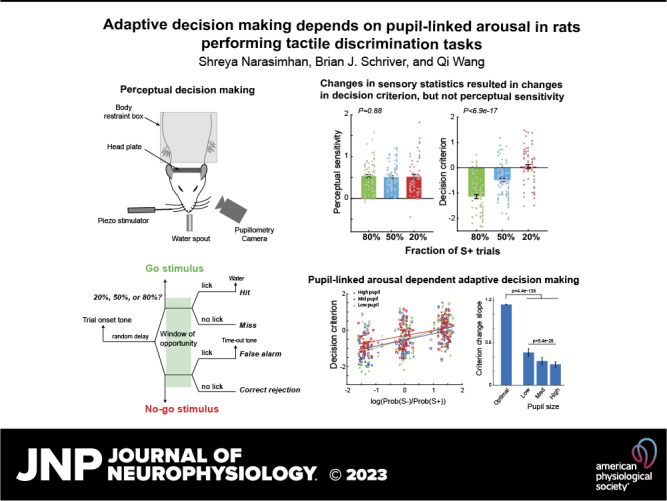

## INTRODUCTION

Adaptive behavior is essential for animals to survive in an ever-varying environment. In perceptual decision-making tasks, sensory information is accumulated over time in the central nervous system, eventually leading to a decision to choose one of the alternatives and generating motor commands to indicate the animal’s choice (1–6). The perceptual decision-making process is shaped by many factors, including brain state, the gain/loss of each possible decision, motivation, task engagement, and external sensory environment (7–19). For example, Waiblinger et al. (20) reported that, in a tactile detection task, rats adjusted their behavioral strategy to maintain a constant payoff in response to changes in the probabilistic distribution of whisker deflection amplitudes.

Multiple neuromodulatory systems may exert heavy influences on the cognitive control of adaptive decision-making (21, 22). Previous work has suggested that tonic activation of the locus coeruleus-norepinephrine (LC-NE) system plays a critical role in regulating decision-making processes with regard to exploring alternatives or exploiting current resources based on the uncertainty of available information (23, 24). On the contrary, phasic activation of the LC-NE system is thought to reset functional networks, and therefore facilitate their reorganization to enable behavioral adaptation in decision-making (25). It has been postulated that the activity of the cholinergic system is related to expected uncertainty (24). The cholinergic system exerts influences on decision-making possibly through the widespread ascending projections from the basal forebrain to the cortex (26–31). Pharmacologically blocking muscarinic receptors suppressed risky decision-making in rats performing a gambling task (32). Nonluminance-mediated changes in pupil size have been successfully used to track rapid fluctuations in cortical state (33, 34) and, thus, are considered as a noninvasive readout of activation of central arousal circuits related to pupil size (i.e., pupil-linked arousal system). Although the exact neural circuitry mediating pupil-linked arousal remains not fully understood, several lines of evidence suggest that multiple neuromodulatory systems, including the LC-NE and cholinergic systems, contribute to pupil-linked arousal to different extents (35–37). However, how adaptive decision-making in response to a varying sensory environment depends on pupil-linked arousal remains unclear.

To address this question, we systematically manipulated the statistics of sensory environment in rats performing a perceptual decision-making task. The fraction of trials in which the Go stimulus was presented (i.e., Go stimulus trials) was randomly selected from 0.8, 0.5, or 0.2 for each session. Our data indicated that the animals adjusted their behavior in response to the changes in the sensory environment in an attempt to maximize water rewards. Interestingly, analysis within the signal detection theory framework revealed that the perceptual sensitivity did not vary across the three paradigms. However, the decision criterion increased as the fraction of Go stimulus trials decreased. Simulation results confirmed that to maximize water rewards, the decision criterion ought to increase if the fraction of Go stimulus trials decreases. Therefore, the observed changes in decision criterion in the animals are likely to reflect adaptive decision-making. By comparing the changes in decision criterion at different pupil-linked arousal levels, we found that the slope of increase in decision criterion across the three paradigms with respect to the decrease in the fraction of Go stimulus trials was closer to the optimal slope during low pupil-linked arousal periods. Together, our results demonstrated that the rats were capable of adapting their decision-making to maximize rewards in response to changes in the sensory environment. Moreover, this adaptive behavior in perceptual decision-making was dependent on pupil-linked arousal.

## MATERIALS AND METHODS

All experimental procedures were approved by the Columbia University Institutional Animal Care and Use Committee and were conducted in compliance with NIH guidelines. Behavioral studies were conducted using five female rats (3 Long Evans and 2 Sprague–Dawley, Charles River Laboratories, Wilmington, MA; ∼225–275 g at the time of implantation). Animals were single-housed after implantation in a dedicated housing facility, which maintained a 12-h light and dark cycle.

### Surgical Implantation

The rodent surgery and implantation of headplates have been described in detail previously (5, 38–40). In brief, in aseptic surgeries, anesthesia was induced with a ketamine/xylazine cocktail (80/5 mg/kg, ip) or isoflurane (1–3% with a nose cone). Ophthalmic ointment was applied to the eyes throughout the surgery to prevent cornea drying. After the scalp was shaved and the hair was removed with depilatory cream, animals were placed in a stereotaxic device using nonpenetrating ear bars (David Kopf Instruments, Tujunga, CA). Buprenorphine (buprenex, 0.03 mg/kg, sc) was administered as an analgesic, and Ringers solution (2 mL, sc) was also administered to prevent dehydration. After exposing and cleaning the skull, 8–10 burr holes were drilled in the skull, and stainless steel screws (0–80 thread, McMaster Carr, Robbinsville, NJ) were inserted to anchor a headplate (14, 41). The wound was then closed with surgical sutures and treated with antibiotic ointment. Antibiotics (Baytril, 5 mg/kg sc) and extra analgesics (ketoprofen, 5 mg/kg sc) were administered for 5 days postoperatively to minimize the risk of infection. The animals began water restriction and subsequent training following approximately 10 days of recovery from implantation surgery.

### Behavioral Procedures

#### Behavioral apparatus.

All behavioral training was conducted in a standard sound and light attenuation chamber (Med Associates, St. Albans, VT). During training, the animals were head-fixed with a custom-made apparatus, in which two pneumatic cylinders on either side of the head were fixed with ball bearings aligned with grooves in the headplate to hold the animals’ heads (14, 42). A 1-mL syringe body, which served to deliver water, was mounted to a flexible beam and placed directly in front of the animal. A piezoelectric force sensor was bonded to the flexible beam to measure voltage swings resulting from animals licking the syringe. The sensor’s output was sampled by a data acquisition (DAQ) card (PCI-6259, National Instruments, Dallas, TX) at 1 kHz.

Precise tactile stimuli were delivered via a multilayer piezoelectric bending actuator (PL140; Physik Instrumente, Germany) driven by a high-voltage amplifier (OPA452; Texas Instruments, Dallas, TX). Whiskers were placed in a short glass capillary pipette approximately 15 mm long with an outer diameter of 1 mm and an inner diameter of 0.5 mm (A-M Systems, Carlsborg, WA). The pipette was bonded to the end of the piezo actuator and placed 8 mm away from the right snout. The whisker that received tactile stimuli was chosen with respect to its thickness between C2, C3, and D2, and the same chosen whisker was used in all behavioral sessions for each animal. The chosen whisker was slightly trimmed to facilitate the insertion into the pipette.

To mask possible auditory cues, a buzzer (bandwidth: 16 Hz–10 kHz) delivering white noise-masking sound was placed next to the whisker stimulator. Onset tone (6 kHz), reward tone (3 kHz), and timeout tones (16.5 kHz) were delivered by a speaker installed in the chamber. Animals were remotely monitored with a CCD camera, and an infrared LED was placed in the chamber for illumination during the task. Control of the behavioral task and sampling of animals’ behavioral responses were performed by custom-programmed software running on a MATLAB xPC target real-time system (MathWorks, Natick, MA). All behavioral data were sampled at 1 kHz and logged for offline analyses.

#### Tactile stimulus.

Whisker stimuli used were sinusoidal waveforms of 8 Hz and 4 Hz (0.5 s, 1 mm amplitude), with the 8 Hz stimulus randomly assigned as the Go stimulus and the 4 Hz stimulus as the No-go stimulus. The probability of the Go stimulus being presented was designated as either 80%, 50%, or 20% for each session.

#### Pupillometry recording.

Recording of the pupil contralateral to the whisker deflection was made using a custom-made pupillometry system (43), which was triggered at 20 Hz by the xPC target real-time system (MathWorks, MA) that controlled the behavioral task. At the beginning of each behavioral session, the ambient luminance in the behavioral chamber was adjusted to make the animal’s pupil size to be at an intermediate level, resulting in similar mean pupil sizes and pupil fluctuations across the three paradigms (Supplemental Fig. S1: https://doi.org/10.6084/m9.figshare.24520669.v1). Pupil images were streamed to a high-speed solid-state drive for offline analysis. To extract pupil size, the pupil contour was segmented using the DeepLabCut toolbox (45). A training set consisting of 200 frames recorded across different sessions was selected. Each frame had 12 evenly distributed points labeled surrounding the pupil manually, and the images were cropped to enable a higher training accuracy. The ResNet50 deep network was used to analyze the video clips from all sessions after the training. The automatically labeled points were fit with circular regression and the pupil size was computed as the area bounded by the contour. 5% of all images were randomly selected for inspection to validate the accuracy of the software. Pupil sizes during blinks were interpolated with values before and after blinks (43, 46). The pupil size was low-pass filtered with a fourth-order noncausal filter with a cutoff frequency of 3.5 Hz.

#### Training and the Go/No-go discrimination task.

Water deprivation schedule and procedures of head-fixation habituation were the same as our previous work (5, 14, 18). Briefly, restriction of access to water was used to motivate animals during the tasks. However, during the behavioral task, correct responses to a Go stimulus were rewarded with ∼60 μL aliquots of water. Because the number of possible rewarding trials (i.e., Go stimulus trials) was different across the three paradigms, supplemental water was given before returning the animals to the animal facility to ensure their daily water intakes were the same across all training days. The weight of the animals was measured and logged immediately after the task.

**Figure 1.**
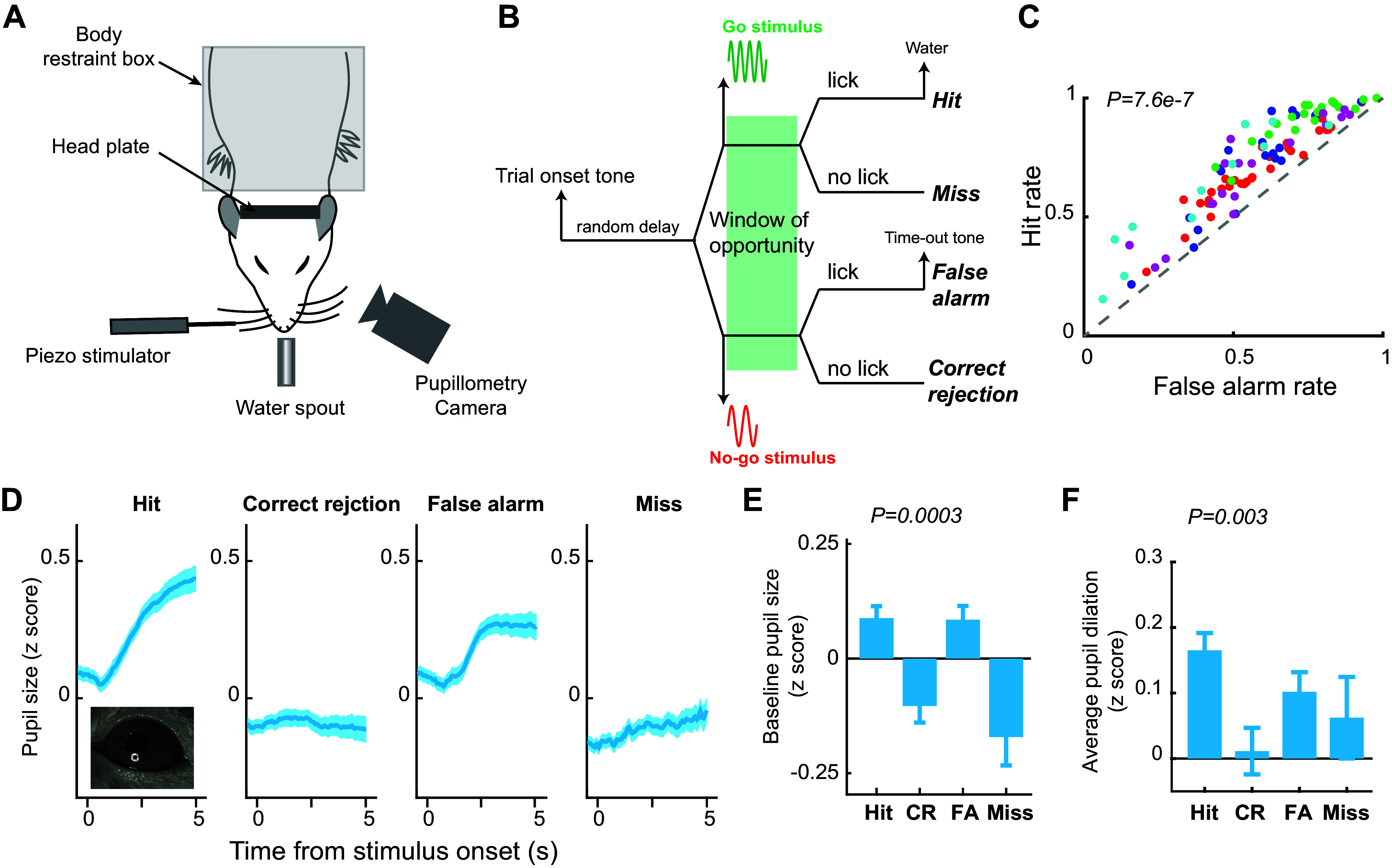
Behavioral performance and pupil dynamics during a tactile decision-making task*. A*: experimental setup. *B:* the diagram of a Go/No-go perceptual decision-making task. *C*: hit rate was significantly higher than false alarm rate in sessions where the probability of the Go stimulus is 50%. Student’s *t* test. Each color denotes an animal. 103 sessions from five animals. *D*: pupil dynamics around stimulus presentation associated with the four behavioral outcomes. *E*: baseline pupil size associated with the four behavioral outcomes. ANOVA test. *F*: pupil dilation associated with the four behavioral outcomes. ANOVA test. 56 sessions from five animals.

The onset of each trial was indicated by a brief “trial onset tone” (300 ms, 6 kHz), followed by a random delay (1 to 3.5 s uniform distribution) (Fig. 1*B*). To discourage the animal from impulsively licking, the last 1 s of the waiting period was a designated “no lick” period, during which any premature licks would result in an additional delay in stimulus presentation pulled from a 1–2.5 s uniform distribution (38, 47). Since the delay of stimulus presentation resulting from premature licks could be repeated indefinitely, our rats usually learned to suppress their impulsive licking during the early stage of behavioral training. The stimulus for each trial could be either a Go stimulus or a No-go stimulus, but the fraction of Go stimulus trials was randomly selected from 0.8, 0.5, and 0.2 for each session, resulting in three behavioral paradigms. Licking within a window of opportunity (1.3 s) following a Go stimulus resulted in a brief “reward tone” (300 ms, 3 kHz) accompanied by a water reward, whereas licking within the window of opportunity following a No-go stimulus triggered a “timeout tone” (5 s, 16.5 kHz) which began a 10-s timeout period. Correct rejection and miss behavioral outcomes were neither rewarded nor penalized. A 6-s inter-trial period followed the end of the window of opportunity for correct rejection and miss trials, water reward for hit trials, and timeout period for false alarm trials. Across all five animals, 260 sessions were performed and 70,516 trials were recorded. Pupillometry was recorded in 165 sessions.

### Data Analysis

All data analyses were first conducted in individual sessions. Grand averages and standard errors of means were then calculated across sessions for analysis and presentation. For each session, the first 20 trials were excluded due to the time required to adjust the pupillometry camera.

#### Behavioral performance.

Response probabilities for each session were calculated as the hit rate (HR, i.e., number of hit trials/number of S+ trials) and false alarm rate (FAR, i.e., number of false alarm trials/number of S− trials). These were used to calculate perceptual sensitivity (*d*′) and decision criterion as:

$$d\prime =\Psi^{-1}\left(Hit\;rate\right)-\Psi^{-1}\left(FA\;rate\right),$$

$$Criterion=-(\Psi^{-1}\left(Hit\;rate\right)+\Psi^{-1}\left(FA\;rate\right))/2,$$

where Ψ^−1^ is the inverse of the cumulative Gaussian distribution.

For analyzing response probabilities, perceptual sensitivity, and decision criterion versus percent of maximum baseline, each session’s baseline range was first computed and then evenly broken into 20 bins, each trial was sorted into one of the bins, and HR, FAR, *d*′, and criterion were calculated for each bin. The log-linear approach was utilized to allow for calculating *d*′ and criterion in bins where HR or FAR equaled 1 or 0, where 0.5 was added to the number of hits and FAs and 1 was added to the number of S+ and S− presentations before calculating HR and FAR (48).

Reaction times were computed as the time from stimulus onset, which is when the window of opportunity began, until the first lick response within the window of opportunity. Reaction times were only computed when a response was logged within the window of opportunity, i.e., for hit and false alarm trials, but not miss or correct rejection trials.

#### Pupil dynamics.

Pupil sizes were first *Z*-scored for each session before further analyses. Pupil sizes were aligned by stimulus onset. Stereotypical pupil responses for each behavioral outcome were calculated as the average pupil size at each time point 0.5 s preceding stimulus onset to 5 s following stimulus onset. Average baselines and dilations were calculated from these averaged stereotypical responses for each behavioral outcome for each session. Baseline pupil sizes were computed as the average of the pupil sizes in the 0.5 s preceding the stimulus, whereas dilations were calculated as the mean value of the pupil sizes from stimulus onset to 5 s post-stimulus onset, minus the pupil baseline. To calculate the percent of maximum pupil baseline, all baselines were normalized for each session.

$$\text{\% }\mathrm{of}\;Maximum\;Pupil\;Baseline=\;\frac{Pupil\;Baseline_{t}-Pupil\;Baseline_{minimum}}{Pupil\;Baseline_{maximum}-Pupil\;Baseline_{minimum}}.$$

Trials were considered in low, medium, or high pupil-linked arousal level if their baseline pupil size was within <33%, 33%–66%, or >66% of the maximum baseline pupil size of the session.

##### Simulation to determine optimal decision criterion.

To determine the optimal decision criterion, we simulated the behavior of rats with different decision criterion in the three paradigms based on the signal detection theory and computed water reward per unit time for each decision criterion in each paradigm. For each simulated session, the probability of S+ trials was set at either 20%, 50%, or 80%. For a given decision criterion, on an S+ trial, a random number was drawn from a normal distribution with mean = 0.52, which is the mean perceptual sensitivity across the three paradigms, and variance = 1. If the random number was greater than the decision criterion, a hit was logged. Otherwise, a miss was logged. For a No-go trial, a random number was drawn for a normal distribution with a mean of 0 and a variance of 1. Either a false alarm or correct rejection was logged, depending on whether the random number was greater than the decision criterion. The duration of a hit trial was composed of a random waiting period (from a 1–3.5 s uniform distribution), a mean response time, and a 6-s inter-trial interval, whereas the duration of a miss or correction rejection trial is composed of a random waiting period (from a 1–3.5 s uniform distribution), a 1.3 s of window of opportunity and a 6-s inter-trial interval. The duration of a false alarm trial is the sum of a random waiting period (from a 1–3.5 s uniform distribution), a mean response time, a 10 s timeout, and a 6-s inter-trial interval. For each paradigm, we simulated 15,000 trials for each decision criterion, and the decision criterion leading to the maximal water reward per unit time was considered the optimal decision criterion. Note that water rewards resulted only from hit responses. We repeated the simulation 20 times to estimate the variance of the optimal decision criterion for each paradigm.

##### HDDM modeling.

Hierarchical drift diffusion models (HDDMs) were used to quantify possible differences in the parameters of decision-making across the three behavioral paradigms (49). We compared four DDM models with various parameter constraints (3, 5, 50). The first model assumed an unbiased starting point, i.e., the start point is the middle of the decision boundary 0.5a or *z* = 0.5 (Fig. 6*A*). For both Go and No-go conditions, the absolute value of the drift rate is the same. The second model assumed that the starting point can be biased, but that the absolute value of the drift rate is the same for both Go and No-go conditions. The third model assumed an unbiased starting point just like the first model, but the drift rate was allowed to be different between the Go and No-go conditions. The fourth model assumed biased starting points and different drift rates for the Go and No-go conditions.

To minimize the risk of overfitting, we calculated the deviance information criterion (DIC) value for each model. Because DIC measure is a tradeoff between goodness of fit and number of free parameters for Bayesian models, we only considered the model with the least DIC as the optimal fit. Each parameter of the model had three group-level distributions corresponding to the three behavioral paradigms and individual-level distributions for each animal.

## RESULTS

To test how animals adaptively change their behavior in perceptual decision-making tasks in response to changes in a sensory environment, we trained head-fixed rats to perform tactile decision-making tasks using a Go/No-go discrimination paradigm (Fig. 1*A*) (14, 18). In these tasks, the rats were required to make decisions to respond or withhold response after a tactile stimulus was presented (Fig. 1*B*). In the initial training sessions, Go stimulus (S+ stimulus, 0.5 s 8 Hz whisker stimulation), which the animal was trained to respond to for rewards, was randomly presented in 50% of trials, whereas No-go stimulus (S− stimulus, 0.5 s 4 Hz whisker stimulation), to which the animal was trained to withhold response to avoid time-out, was presented in the rest of the trials. Animals had significantly higher response probability to S+ than to S− stimulus in these sessions (0.72 ± 0.019 vs. 0.58 ± 0.02, *P* < 7.6e-07, paired *t* test, means ± SE; error bars indicate 1 SE unless otherwise noted, Fig. 1*C*), indicating that the animal understood the requirement. Moreover, consistent with our previous work, the pupil size of the rats fluctuated throughout the sessions. The pupil dynamics around stimulus presentation were different across the four possible behavioral outcomes (i.e., hit, correct rejection, false alarm, miss) (Fig. 1, *D* and *E*). Baseline pupil size before stimulus onset was higher for hit and false alarm trials than for correct rejection and miss trials (hit: 0.08 ± 0.023; correct rejection: −0.085 ± 0.036, false alarm: 0.074 ± 0.027, miss: −0.14 ± 0.07, *P* = 0.0003, ANOVA test). Task-evoked pupil dilation was largest for hit trials, followed by false alarm trials, miss trials, and correct rejection trials (hit: 0.156 ± 0.015; false alarm: 0.096 ± 0.018; miss: 0.07 ± 0.034; correct rejection: 0.0095 ± 0.022; *P* = 0.003; ANOVA test).

**Figure 2.**
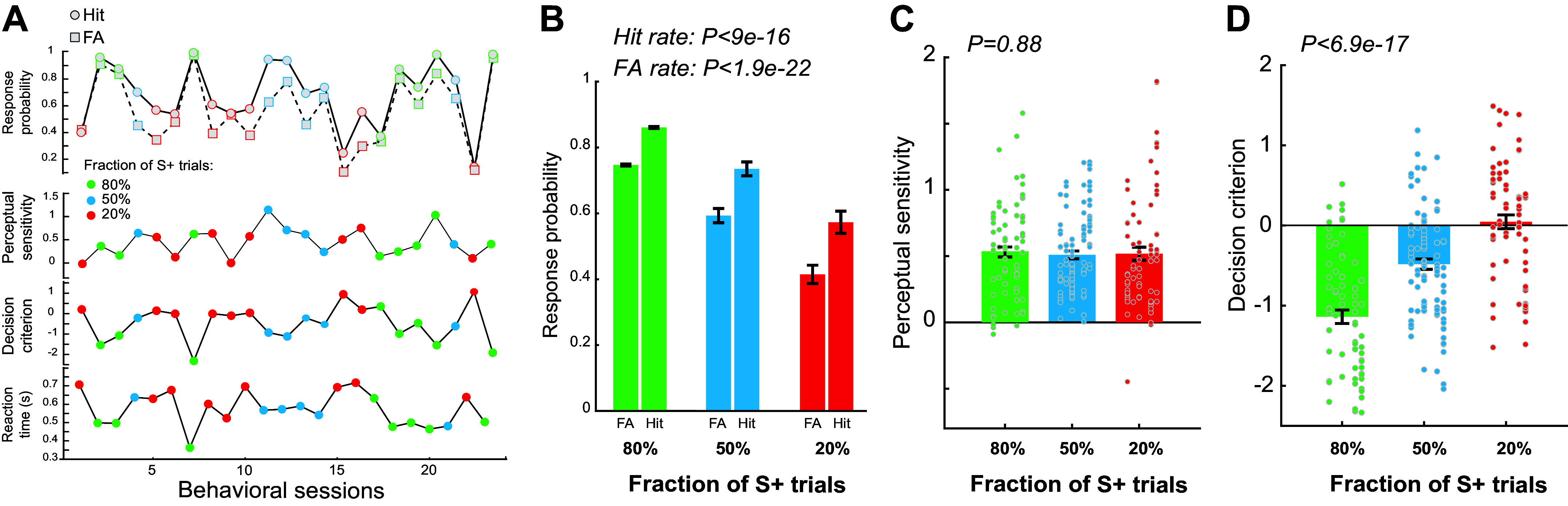
Adaptive behavior in response to change in the statistics of sensory environment. *A*: example adaptive behavior in response to change in the probability of S+ trials. *B*: response probability across the three paradigms. ANOVA test. 260 sessions from five animals. *C*: perceptual sensitivity remained the same across the three paradigms. ANOVA test. Each column of dots represents an animal. *D*: decision criterion significantly increased as the probability of S+ trials decreased. Each column of dots represents an animal. ANOVA test. 260 sessions from five animals.

To test whether the animals adaptively changed their behavior in response to changes in sensory environment, we systematically manipulated the statistics of sensory signals. In our experiments, we used three paradigms in which fractions of S+ trials, i.e., trials on which S+ stimulus was presented, were set at 20%, 50%, and 80%. Each session was randomly assigned with one paradigm and its corresponding fraction of S+ trials. We found that animals adaptively changed their response rate in response to changes in the fraction of S+ trials for each session (Fig. 2*A*). In general, both hit rate and false alarm rate decreased as the fraction of S+ trials decreased from 80% to 20% (0.8574 ± 0.0021 vs. 0.723 ± 0.002 vs. 0.552 ± 0.003, *P* < 9e-16, ANOVA test; false alarm rate: 0.7622 ± 0.0025 vs. 0.578 ± 0.002 vs. 0.398 ± 0.003, *P* < 1.9e-22 ANOVA test) (Fig. 2*B*). Using the signal detection theory framework (39, 41, 51, 52), we found that the perceptual sensitivity of the animals was not affected by the changes in fraction of S+ trials, as there was no significant difference across the three paradigms with different fractions of S+ trials (0.536 ± 0.042 vs. 0.510 ± 0.029 vs. 0.518 ± 0.045, *P* = 0.88, ANOVA test) (Fig. 2*C*). However, we found that the animals systematically adjusted their decision criterion from more liberal (i.e., more negative decision criterion) to more conservative (i.e., less negative decision criterion) in responses to the decrease in the fraction of S+ trials (−1.139 ± 0.08 vs. −0.483 ± 0.064 vs. 0.045 ± 0.085, *P* < 3.1e-20, ANOVA test) (Fig. 2*D*). These phenomena held for both strains and individual rats: all rats exhibited consistent increase in decision criterion along with decrease in the fraction of S+ trials while the changes in their perceptual sensitivity were mixed (Supplemental Fig. S2: https://doi.org/10.6084/m9.figshare.24520669.v1).

**Figure 3.**
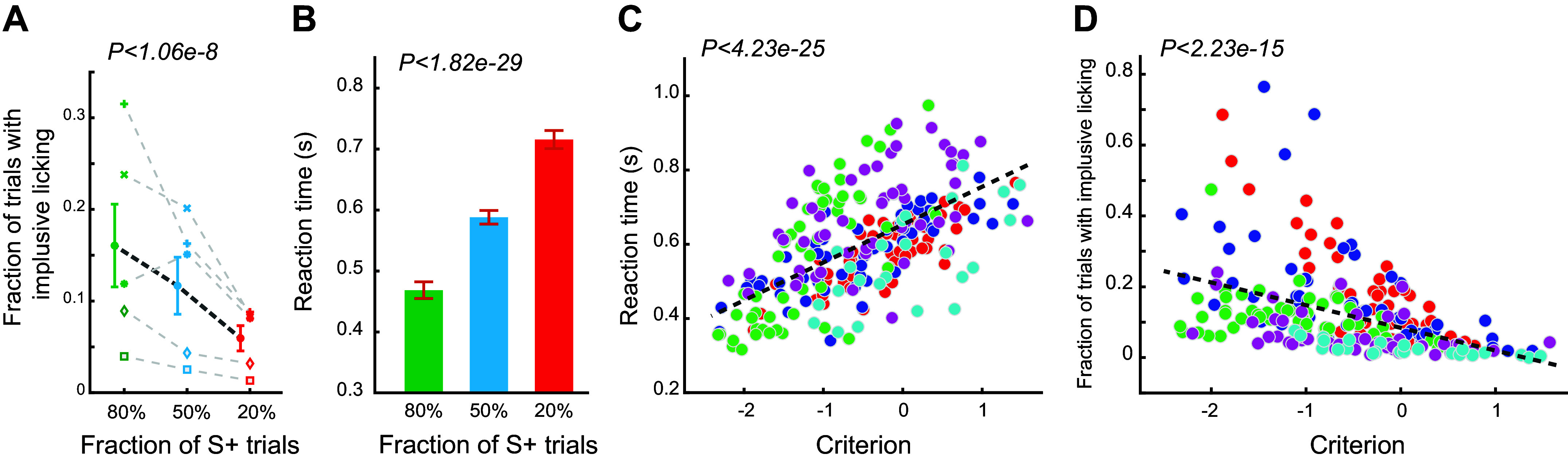
Changes in impulsive licking rate and reaction time in the three paradigms. *A*: impulsive licking rate decreased along with the decrease in probability of S+ trials. ANOVA test. 260 sessions from five animals. *B*: reaction time significantly increased as the probability of S+ trials decreased. ANOVA test. 260 sessions from five animals. *C*: reaction time is positively correlated with decision criterion. Each color denotes an animal. 260 sessions from five animals. *D*: impulsive licking rate is negatively correlated with decision criterion. Each color denotes an animal. 260 sessions from five animals.

Because animals only received water rewards on hit trials during the task, animals could accumulate less water intake in sessions with the fraction of S+ trials being 0.2, compared with the other two paradigms. Although we gave each animal supplemental water right before we returned them to the animal facility on each training day to ensure they had the same daily water intake across sessions with different paradigms, it was still possible that the difference in level of thirst during the task may have resulted in the changes in the response probability to both Go and No-go stimuli. However, if the animals were thirstier, it would be plausible that they should have high response rates in an attempt to get more water. However, this was contrary to what we experimentally observed (Fig. 2*B*). To further rule out this possibility, we calculated the percent of trials in which the animal impulsively licked (i.e., licked between trial onset tone and stimulus presentation). We reasoned that if animals were thirstier in sessions where 20% of the trials were S+ trials, they would tend to lick more impulsively, leading to a higher impulsive licking rate. However, our data suggested that this was not the case. The fraction of impulsive licking trials was significantly smaller for sessions where 20% of the trials were S+ trials, compared with the other two paradigms (0.174 ± 0.019 vs. 0.134 ± 0.0106 vs. 0.062 ± 0.0055, *P* < 1.06e-8, ANOVA test) (Fig. 3*A*), suggesting that the changes in response probability resulted from cognitive processing in responses to changes in probability of S+ trials, rather than the level of thirst. Supporting this notion, we found that the reaction time monotonically increased with the decrease in fraction of S+ trials (0.4838 ± 0.012 s vs. 0.594 ± 0.011 s vs. 0.702 ± 0.011 s, *P* < 1.82e-29, ANOVA test) (Fig. 3*B*). Furthermore, decision criterion was positively correlated with reaction time (*P* < 4.23e-25) (Fig. 3*C*). As we expected, our data showed that decision criterion was negatively correlated with percent of impulsive licking trials (*P* < 2.23e-15) (Fig. 3*D*). Taken together, these results suggested that the adaptive behavior of the animals that we observed in our experiments was due to higher-level cognitive processing of the statistics of sensory environment, rather than low-level physiological needs such as thirst.

**Figure 4.**
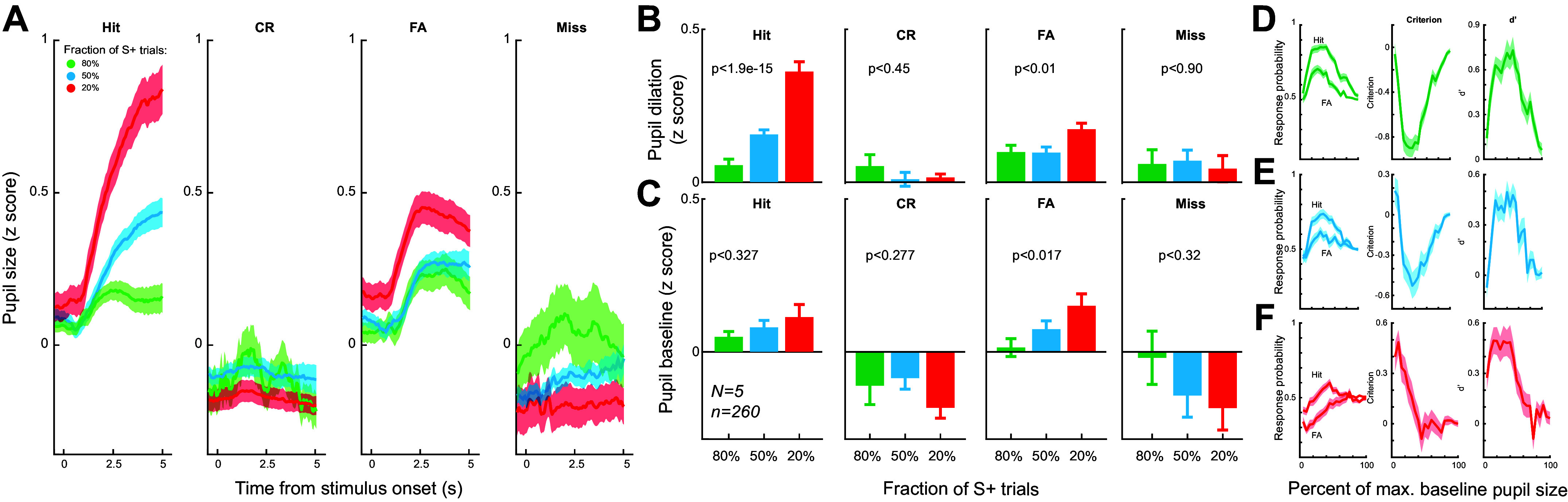
Pupil dynamics in the three paradigms. *A*: pupil dynamics around stimulus presentation associated with the four behavioral outcomes in the three paradigms. 165 sessions from five animals. *B:* baseline pupil size associated with the four behavioral outcomes in the three paradigms. ANOVA test. 165 sessions from five animals. *C*: pupil size associated with the four behavioral outcomes in the three paradigms. ANOVA test. 165 sessions from five animals. *D–F*: the relationship between response probability/decision criterion/perceptual sensitivity and baseline pupil size in the three paradigms. 165 sessions from five animals

We have previously shown that perceptual decision-making depended on pupil-linked arousal (5). We then examine if pupil dynamics were different during adaptive decision-making across the three paradigms. Indeed, the pupil dynamics around stimulus presentation were significantly different between the three paradigms for the four behavioral outcomes (Fig. 4*A*). We found that there was a dramatic difference in task-evoked pupil dilation between the three paradigms for hit trials (0.0554 ± 0.02 vs. 0.156 ± 0.015 vs. 0.36 ± 0.03, *P* < 1.9e-15, ANOVA test), and to a lesser degree for false alarm trials (0.098 ± 0.022 vs. 0.096 ± 0.018 vs. 0.173 ± 0.0195, *P* < 0.01, ANOVA test), but not for the other two behavioral outcomes (correct rejection: 0.0525 ± 0.037 vs. 0.009 ± 0.028 vs. 0.154 ± 0.014, *P* = 0.45, ANOVA test; miss: 0.0595 ± 0.0466 vs. 0.07 ± 0.035 vs. 0.04 ± 0.043, *P* = 0.90, ANOVA test) (Fig. 4*B*). There was a significant change in baseline pupil size between the three paradigms for false alarm trials (0.0142 ± 0.029 vs. 0.074 ± 0.07 vs. 0.15 ± 0.04, *P* < 0.017, ANOVA test), whereas there was not a significant difference in baseline pupil size between the three paradigms for the other three behavioral outcomes (hit: 0.049 ± 0.017 vs. 0.08 ± 0.023 vs. 0.113 ± 0.04, *P* = 0.32, ANOVA test; correct rejection: −0.11 ± 0.062 vs. −0.085 ± 0.036 vs. −0.183 ± 0.034, *P* = 0.27, ANOVA test; miss: −0.0194 ± 0.087 vs. −0.1426 ± 0.007 vs. −0.183 ± 0.072, *P* = 0.32, ANOVA test) (Fig. 4*C*). Interestingly, in paradigms with the fraction of S+ trials being 0.8 and 0.5, there was a profound inverted-U or U-shaped relationship between baseline pupil size and hit/false alarm rates, decision criterion, and perceptual sensitivity (Fig. 4, *D* and *E*). However, this inverted-U or U-shaped relationship between baseline pupil size and hit/false alarm rates, decision criterion, and perceptual sensitivity was less conspicuous for the paradigm in which the fraction of S+ trials is 0.2 (Fig. 4*F*).

**Figure 5.**
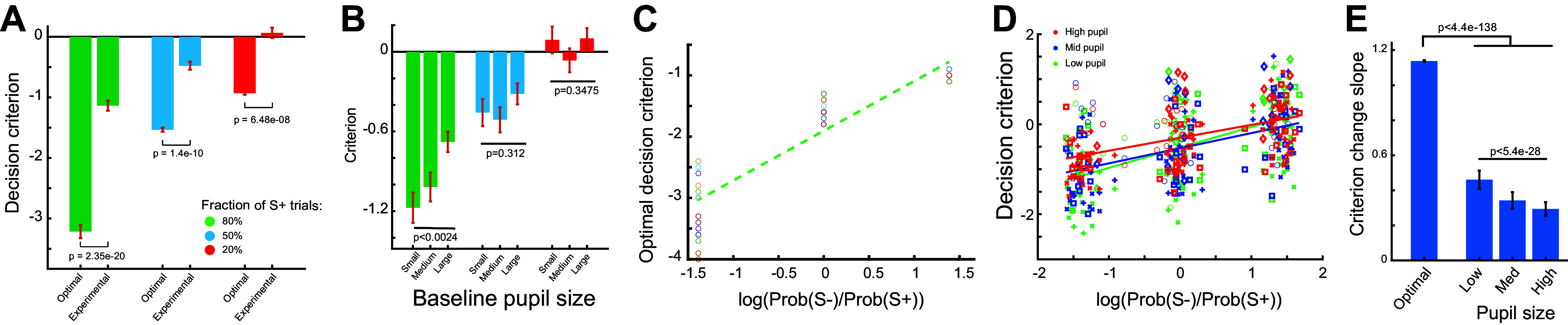
Optimal adjustment of decision criterion is dependent on pupil size. *A*: optimal and experimentally observed decision criterion in the three paradigms. Student’s *t* test. 260 sessions from five animals. *B*: decision criterion of the animals at different pupil-linked arousal levels. ANOVA test. 260 sessions from five animals. *C*: optimal adaptation of decision criterion in response to changes in probability of S+ trials. *D*: adaptation of decision criterion in response to changes in probability of S+ trials in the animals at different pupil-linked arousal levels. Each symbol denotes an animal. 165 sessions from five animals. *E*: optimal and experimentally observed adaptation of decision criterion in response to changes in probability of S+ trials. ANOVA test.

How were the pupil dynamics related to the adaptive behavior? Our data demonstrated that task-evoked pupil dilations were different across the three paradigms and that the animals mostly adjusted their decision criterion while maintaining the same perceptual sensitivity across the three paradigms. To determine the optimal decision criterion for each paradigm, we simulated the water reward per unit time with different decision criteria for each paradigm using our experimental parameters. If a decision criterion was too negative, the animals would be liberal in making Go decisions. Consequently, they would encounter many false alarms, and thus a substantial portion of the task would be in the time-out period. On the contrary, if the animals were too conservative in making Go decisions and set the decision criterion to be a large positive value, the animals would falsely reject many S+ stimuli, resulting in a low hit rate and less water intake throughout the task period. Our simulation results indicated that the optimal decision criterion was significantly smaller than the ones that we observed experimentally. For the paradigm with fraction of S+ trials being 0.8, the optimal decision criterion and observed decision criterion were −3.215 ± 0.107 versus −1.137 ± 0.084 (*P* < 0.2.35e-20, *t* test). Similarly, the optimal and observed decision criterion were −1.535 ± 0.033 versus −0.479 ± 0.065 (*P* < 1.43e-10, *t* test) and −0.93 ± 0.0275 versus 0.0636 ± 0.086 (*P* < 6.5e-08, *t* test) for the two other paradigms, respectively (Fig. 5*A*). We further examined if the decision criterion was dependent on pupil size within each paradigm. We found that in the paradigm where 80% of trials were S+ trials, the decision criterion increased monotonically with baseline pupil size (*P* < 0.0025). However, this trend did not hold for the other two paradigms (Fig. 5*B*).

Although these results indicated that the animals were suboptimal in terms of their decision-making, the adaptive change in decision criterion observed in our experiments was in line with the optimal adjustment of decision criteria. We therefore compared changes in decision criteria across the three paradigms, measured as the slope of decision criterion across the three paradigms, between the optimal decision-making case (i.e., simulation) and the real case (i.e., experiments). We found that the change in optimal decision criteria in response to changes in paradigms, i.e., the slope of optimal decision criterion across the three paradigms, was much steeper than the experimentally observed slope (1.14 vs. 0.355) (Fig. 5*C*). We further examined if the adjustment of decision criterion in response to changes in sensory environment depended on pupil-linked arousal indexed by pupil size. To this end, we grouped trials of each session into three groups based on the baseline pupil size, and calculated decision criteria for trials within each group (Fig. 5*D*). We found that there was a systematic change in the slope along the baseline pupil size, with the slope being largest during small baseline pupil size (*P* < 5.4e-28, ANOVA test). However, the slopes for all pupil size-dependent groups were significantly smaller than the slope of optimal decision criteria (*P* < 4.4e-138, ANOVA test) (Fig. 5*E*).

**Figure 6.**
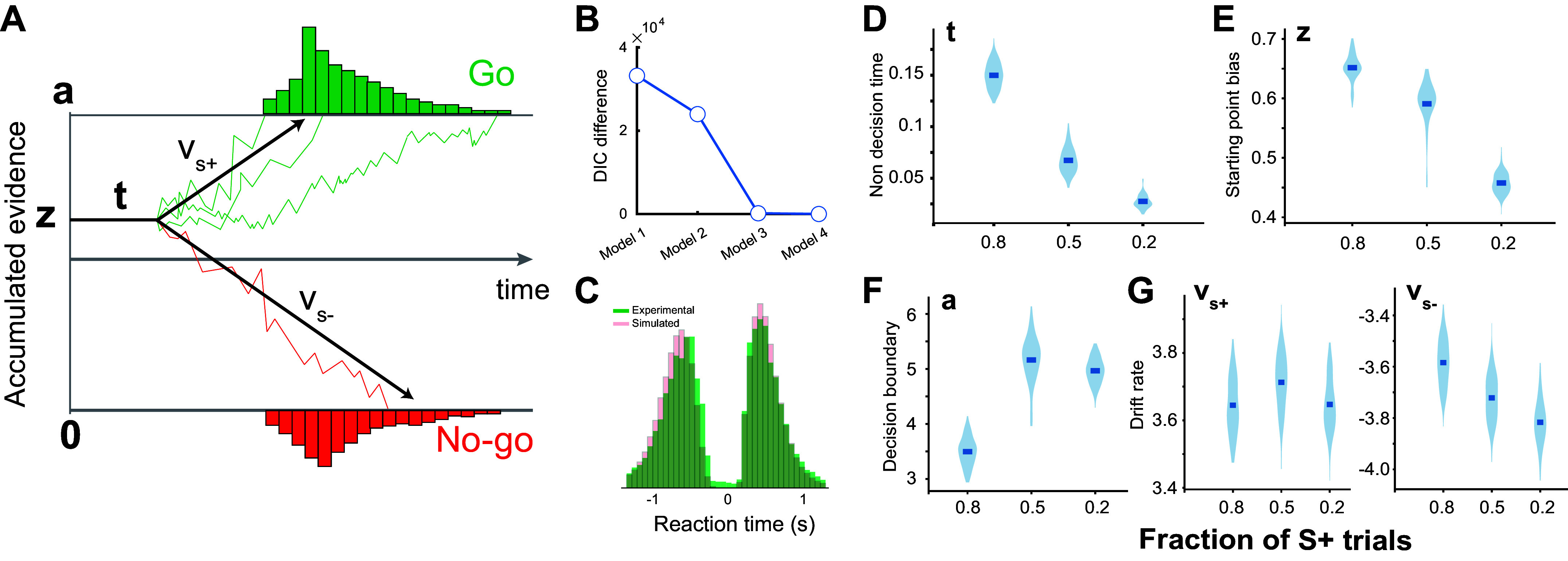
HDDM modeling of perceptual decision-making in the three paradigms. *A*: diagram of the drift diffusion model. *B*: deviance information criterion (DIC) difference between the four alternative models. *C*: reaction time distributions were captured by the HDDM. *D–G*: violin plots showing the posterior estimates of the HDDM parameters for the three paradigms. HDDM, hierarchical drift diffusion model.

We further used the drift diffusion model (DDM) to quantify the extent to which the other parameters of decision-making, including non-decision time, decision boundary, drift rate, and initial bias, were affected by the changes in sensory environment (Fig. 6*A*). To this end, we used a Bayesian approach to estimate the distributions of decision-making parameters at the group level for each paradigm (49), and P_p|D_ was used to refer to the proportion of posteriors from Bayesian inference, supporting the working hypothesis that there was a difference between the paradigms at the group level. We first calculated the deviance information criterion (DIC) value of the four variants of the hierarchical DDM for our behavioral data (see methods). Since DIC balances between a goodness-of-fit of the model and additional free model parameters, we used the model with the lowest DIC value (Fig. 6*B*). This model generated a similar distribution of reaction times to those measured experimentally (Fig. 6*C*). HDDM results suggested a significant difference in non-decision time and initial bias among the three paradigms (P_p|D_ ≈ 1) (Fig. 6, *D* and *E*). However, for the decision boundary, there was only a significant difference between the paradigm with 80% S+ trials and both paradigms with 50% and 20% S+ trials (P_p|D_ ≈ 1), and there was no difference between the paradigm with 50% S+ trials and the paradigm with 20% S+ trials (P_p|D_ = 0.2) (Fig. 6*F*). We failed to find significant differences in drift rate across the paradigms (P_p|D_ > 0.05) (Fig. 6*G*).

## DISCUSSION

Our previous work investigated how pupil-linked arousal modulates behavioral performance (18) and the extent to which arousal systems indexed by electrocardiograph signals and pupil size differently modulate perceptual behavior (14). The present study was designed to allow us to investigate adaptive behavior in rats performing perceptual decision-making tasks. Previous work has demonstrated adaptive behavior in response to changes in rewards and stimulus properties (9, 20). In this study, we manipulated the probability of Go and No-go stimuli and characterized the animals’ adaptive decision-making at different pupil-linked arousal levels. The results present several novel findings. First, we showed that the animals became more liberal in making a Go decision when the probability of S+ stimulus increased. This behavioral adaptation is in line with the optimal adaptation to maximize rewards during the task (Figs. 2 and 5). Second, task-evoked phasic pupil-linked arousal is higher when the probability of S+ is low (Fig. 4). Finally, adaptive decision-making is dependent on pupil-linked arousal (Fig. 5, *D* and *E*). Consistent with human results (53), our animals performed suboptimally in adjusting their decision criterion in response to varying probabilities of S+ trials. For all three paradigms, the animals could have received more water rewards if they were more liberal in making Go decisions in the behavioral tasks (Fig. 5*A*). One possible explanation could be that the tone indicating the onset of the timeout period became aversive to the animals, increasing the cost of false alarms.

We systematically manipulated the probability of S+ trials, varying from either 20%, 50%, or 80% in the experiments, as a means to probe adaptive decision-making. Uncertainty of sensory inputs and rewards has been shown to impose effects on neural computation and decision-making (19, 24, 54–57). It is important to note that the uncertainty of sensory stimuli is theoretically the same for the two paradigms in which the probability of S+ trial is 80% and 20% in our experiments. If uncertainty plays a critical role in adaptive decision-making and evoking pupil dilation in our experiments, we would expect to see the same adaptive behavior and pupil dynamics in those two paradigms. However, we observed a monotonic increase in both decision criterion and task-evoked pupil dilation across the three paradigms, suggesting that the uncertainty of stimulus is not a primary factor responsible for adaptive decision-making.

What behavioral state did our experimental conditions manipulate in the animals? For the paradigms with a lower probability of S+ trials, we observed a slowdown of reaction times and less impulsive licking. So it is possible that the animals were less engaged or less attentive in the task for these paradigms. Task engagement and attention have been shown to modulate neural representation and cognitive processing in behavioral tasks (8, 15, 58–61), and thus might be an important contributor to adaptive decision-making. However, the animals’ behavioral performance does not support this possibility because there was no significant difference in perceptual sensitivity across the three paradigms. However less engagement and poor attention usually lead to poor performance in perceptual tasks. Moreover, the task-evoked pupil dilation is largest in the paradigm where the probability of S+ trials was 20%, but previous work suggested larger task-evoked pupil dilation during more task-engaged or attentive periods (9, 62). Therefore, the behavioral adaptation during perceptual decision-making in our experiments is unlikely to be primarily due to changes in behavioral states such as attention or engagement in the task. A possibility is that this behavioral adaptation is driven by different internal models involving probabilistic inference and expectation (24, 25, 63). Another explanation of the observed behavioral adaptation would be the motivation associated with each behavioral paradigm as previous work has shown that over-motivated state and under-motivated state resulted in different response probabilities in perceptual tasks (8, 15). The increase in reaction time and decrease in response probability can be interpreted as the animals were under-motivated due to a low chance of obtaining water reward in the behavioral paradigm where the fraction of S+ trials was 0.2. However, the animals were thirstier due to less total water reward in this paradigm as compared with the other two paradigms, and therefore should presumably be more motivated. The cause of the adaptive decision-making is thus likely to involve both mechanisms. Another interesting observation is that the animals used in this study exhibited lower perceptual sensitivities on average than our previous studies despite the fact that the experimental setup used in both studies was almost identical (18). This may be because we randomly switched paradigms between sessions in the study and the animals had to allocate some mental efforts to update internal models during the task, resulting in a poorer perceptual performance. Future work with electrophysiological recordings and manipulations in higher-order brain regions (e.g., the prefrontal cortex or parietal cortex) and neuromodulatory systems will help answer this intriguing question (19, 64).

Our findings provide new evidence that adaptive decision-making is dependent on pupil-linked arousal. Previous work suggested that pupil size is able to reliably index the activation of the LC-NE system, as microstimulation of the LC evoked dramatic dilation of pupil in rats and non-human primates (43, 65) (but also see Ref. 66 for the relationship between LC activity and pupil size in mice). Recent experimental results also demonstrated that pupil size co-varies with cholinergic activity in the brain to a lesser degree. Cholinergic neurons of the basal forebrain are more active during pupil dilation (36). Two-photon imaging revealed a positive correlation between the activation of cortical cholinergic axons and pupil size (37). Recent results showed that phasic stimulation of serotonergic neurons in the dorsal raphe nucleus causes pupil size changes in mice performing a foraging task (35). Therefore, it is likely that the three neuromodulatory systems collectively exert influences on adaptive decision-making in our experiments. It is intriguing for future studies to use selective manipulation to tease apart the contribution that each of the neuromodulatory systems provides to adaptive decision-making.

## DATA AVAILABILITY

All data and code are available upon request.

## SUPPLEMENTAL DATA

10.6084/m9.figshare.24520669.v1 Supplemental Fig. S1 and Fig. S2: https://doi.org/10.6084/m9.figshare.24520669.v1.

## GRANTS

This work was supported by NIH R01MH112267, R01NS119813, R21MH125107, and the Air Force Office of Scientific Research under award number FA9550-22-1-0337.

## DISCLAIMERS

Any opinions, findings, and conclusions or recommendations expressed in this material are those of the authors and do not necessarily reflect the views of the United States Air Force.

## DISCLOSURES

Q.W. is the co-founder of Sharper Sense, a company developing methods of enhancing sensory processing with neural interfaces. None of the other authors has any conflicts of interest, financial or otherwise, to disclose.

## AUTHORS CONTRIBUTIONS

Q.W. conceived and designed research; S.N. and Q.W. performed experiments; S.N. and B.J.S. analyzed data; S.N., B.J.S., and Q.W. interpreted results of experiments; Q.W. prepared figures; Q.W. drafted manuscript; S.N. and Q.W. edited and revised manuscript; S.N., B.J.S., and Q.W. approved final version of manuscript.
